# Unidirectional Wave Propagation in Low-Symmetric Colloidal Photonic-Crystal Heterostructures

**DOI:** 10.3390/nano5010376

**Published:** 2015-03-19

**Authors:** Vassilios Yannopapas

**Affiliations:** Department of Physics, National Technical University of Athens, GR-15780 Athens, Greece; E-Mail: vyannop@mail.ntua.gr; Tel.: +30-210-7721481

**Keywords:** photonic crystals, wave transmission, multiple-scattering

## Abstract

We show theoretically that photonic crystals consisting of colloidal spheres exhibit unidirectional wave propagation and one-way frequency band gaps without breaking time-reversal symmetry via, e.g., the application of an external magnetic field or the use of nonlinear materials. Namely, photonic crystals with low symmetry such as the monoclinic crystal type considered here as well as with unit cells formed by the heterostructure of different photonic crystals show significant unidirectional electromagnetic response. In particular, we show that the use of scatterers with low refractive-index contrast favors the formation of unidirectional frequency gaps which is the optimal route for achieving unidirectional wave propagation.

## 1. Introduction

Photonic computing holds promise for superior processing of information in terms of speed, liability and bandwidth. Key ingredients in a photonic/optical computer are the waveguiding devices which steer light among different photonic components (diodes, optical transistor, optical buffers, *etc.*). Although this is easily accomplished in the microwave regime via conventional metallic waveguides, in the optical regime it still is a subject of intensive research as commercially available optical fibers cannot efficiently bend light around sharp corners at micrometer or even lower dimensions. Perhaps the most robust method for steering and bending light in the micrometer scale are the so-called photonic-crystal fibers [1]. Namely, light is localized efficiently within a dielectric or air core due to the suppression of propagation within the photonic-crystal cladding which exhibits an omnidirectional band gap for the waveguiding frequencies. Device performance in photonic-crystal fibers can be deteriorated by fabrication imperfections which lead to important backscattering of light, especially at sharp edges. A different approach for guiding light in photonic computers lies with the discipline of plasmonics wherein the excitation of surface plasmons in noble-metal components leads to light localization in the nanoscale enabling efficient light steering at very small volumes [2]. The main obstacle in this technology are the inherent losses of noble metals in the optical regime which degrade light guiding at sufficiently long distances.

A recent idea which has been put forward as an alternative to the current technologies for light guiding is the so-called unidirectional or one-way waveguiding, *i.e.*, propagation of light solely along one of the two possible directions in a straight waveguide. A direct consequence of the inhibition of light propagation in the other direction is the complete absence of light backscattering due to fabrication-induced disorder and imperfections or at very sharp guiding edges. This is easily achieved in the GHz regime via introducing time-reversal symmetry breaking in the microwave circuitry with the use of ferromagnetic materials and application of magnetic fields [3,4,5,6,7]. However, in the optical regime, again, it is really challenging to implement such a strategy as the magnetic contribution of ferromagnetic materials at this spectral region is very weak. Artificial structures made of magneto-optically responsive materials can potentially provide a means to achieve unidirectional waveguiding without however success up to now, due to their weak contribution to the overall electromagnetic response of such materials. Although the magneto-optical contribution can be very much enhanced with the insertion of plasmonic materials [8,9,10,11,12,13,14,15,16,17] , experimental evidence of unidirectional waveguiding via magneto-optical structures is still lacking.

On the other hand, there have been alternative approaches so as to achieve unidirectional propagation/guiding without the use of magnetic materials and subsequent application of an external magnetic field. Namely, unidirectional propagation has been reported in nonlinear photonic crystals (PCs) [18,19,20,21], in photonic-crystal heterostructures [22,23,24,25] as well as in 2D PCs with periodically modulated interfaces [26,27,28]. However, in the above cases, although the application of an external magnetic field is no longer needed, the range of frequencies and angles of incidence within which unidirectional propagation occurs is very narrow. Recently we have presented a linear, passive design of a purely dielectric 3D PC which possess unidirectional (one-way) photonic band gaps spanning over wide regions in both the Brillouin zone and EM spectrum [29]. The respective unit cell of the PC possess a degree of chirality while, at the same time, consists of different material scatterers giving rise to unidirectional wave propagation and frequency gaps. In the present work, we proceed further with this PC design and explore its potential for unidirectional light propagation. Namely, we consider PC designs with real materials simulating the response of colloidal photonic-crystal heterostructures. We show, in particular, that the emergence of unidirectional frequency bands and gaps is favored by materials with low refractive-index contrasts wherein the PC frequency bands are described as a perturbation to the free-photon frequency bands of an empty lattice.

## 2. Description of the Photonic-Crystal Heterostructure

**Figure 1:**
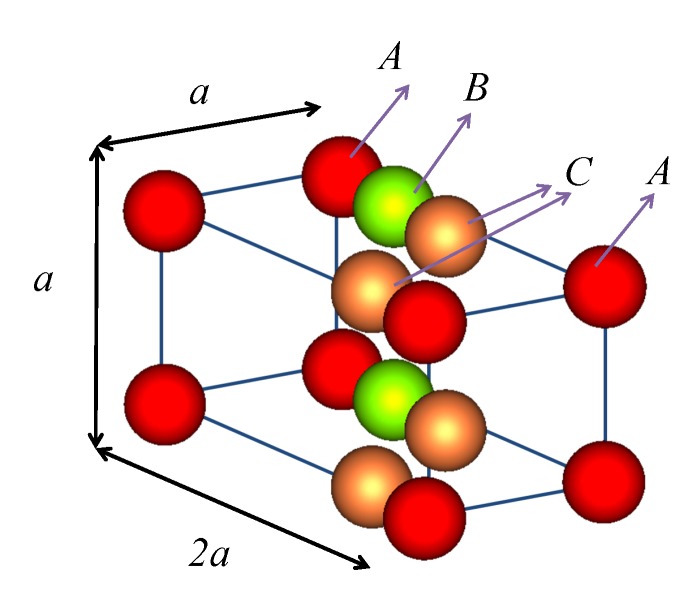
Unit cell of 3D PC with one-way photonic band gaps: monoclinic crystal consisting of four non-primitive planes of spheres parallel to the (001) surface at positions (0, 0, 0), (0, 0, *a*/2), (0, 0, *a*), and (−0.3*a*, 0, 3*a*/2).

We deal with the 3D photonic crystal shown in Figure 1. It can be described as a monoclinic crystal with a four-point basis. The crystal is viewed as a succession of planes of spheres parallel to the *xy*-plane. Each plane possesses the same 2D periodicity defined by the primitive vectors **a**_1_ = (*a*, 0, 0) and **a**_2_ = (0, *a*, 0) [(001) crystallographic surface]. This means that we have a square lattice with lattice vectors **R***_n_* = *n*_1_**a**_1_ + *n*_2_**a**_2_, *n*_1_, *n*_2_ = 0, ±1, ±2, ··· and corresponding reciprocal lattice vectors **g** = *m*_1_**b**_1_ + *m*_2_**b**_2_, *m*_1_, *m*_2_ = 0, ±1, ±2, ··· where **b***_i_* · **a***_j_* = 2πδ*_ij_*, *i*, *j* = 1, 2. A unit layer of the crystal consists of four non-primitive planes of spheres at (0, 0, 0), (0, 0, *a*/2), (0, 0, *a*), and (−0.3*a*, 0, 3*a*/2). All the spheres of the crystal of Figure 1 have the same radius *S* = 0.2*a*. The spheres of the first layer are of type A, of the second type B while the spheres at the third and fourth layers are of type C. The (*n* + 1)-th unit layer is obtained from the *n*-th layer by the primitive translation **a**_3_ = (−0.3*a*, 0, 2*a*). Such a monoclinic PC lattice has only one mirror plane [xz-plane, *i.e.*, (010) crystallographic surface] and is therefore a lattice of low symmetry. We can thus expect that light transmission through a finite slab of such a monoclinic PC lattice would be different for incidence from the two opposite faces of the slab, if the slab is considered as a stack of planes parallel to a crystallographic direction other than the (010). In what follows, the different sphere types correspond to spheres made of different materials. In principle, one-way propagation can also be achieved by having spheres of different size or scatterers of dissimilar shape.

## 3. Calculation Method

The PC considered in the preceding section will be studied in terms of its frequency band structure and light transmission/absorption by employing the layer-multiple-scattering (LMS) method [30,31,32,33,34]. The LMS method is ideally suited for the calculation of the transmission, reflection and absorption coefficients of an EM wave incident on a composite slab consisting of a number of layers which can be either planes of non-overlapping spherical [30,31,32] or nonspherical axisymmetric [33] and of general shape [35] particles or clusters of such [34], with the same 2D periodicity or homogeneous plates. For each plane of particles, the method calculates the full multipole expansion of the total multiply scattered wave field and deduces the corresponding transmission and reflection matrices in the plane-wave basis. The transmission and reflection matrices of the composite slab are evaluated from those of the constituent layers. By imposing periodic boundary conditions one can also obtain the (complex) frequency band structure of an infinite periodic crystal. The method applies equally well to non-absorbing systems and to absorbing ones. Its main advantage over the other existing numerical methods lies in its efficient and reliable treatment of systems containing strongly dispersive materials such as Drude-like and polaritonic materials.

## 4. Results and Discussion

**Figure 2:**
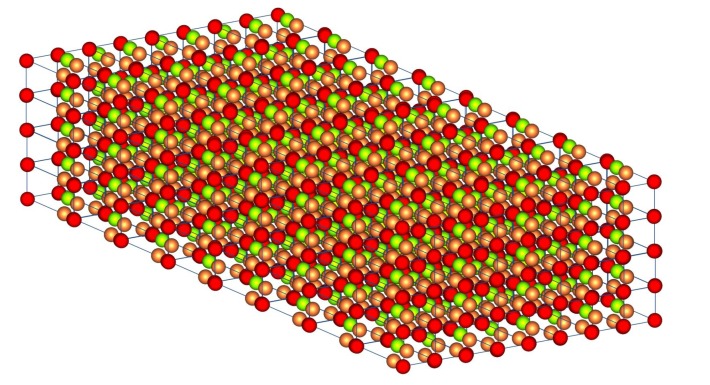
Finite slab of the photonic crystal of Figure 2 consisting of eight unit layers.

In order to assess the asymmetrical transmission of light which would possibly lead to the generation of unidirectional photonic frequency bands/gaps, we calculate the transmittance of light incident on finite slabs of the 3D PC of Figure 1, based on the LMS method analyzed above. In Figure 2 we show a finite slab of the photonic crystals studied here wherein the slab consists of eight unit layers. As type-A spheres we have considered silica SiO_2_ (ϵ = 2.1) spheres, as type-B polystyrene (ϵ = 2.6) spheres and as type-C silicon (ϵ = 11.9) (Si) spheres. All three types of materials have been the basis for the fabrication of colloidal photonic crystals of nanospheres (for a review see [36]).

**Figure 3:**
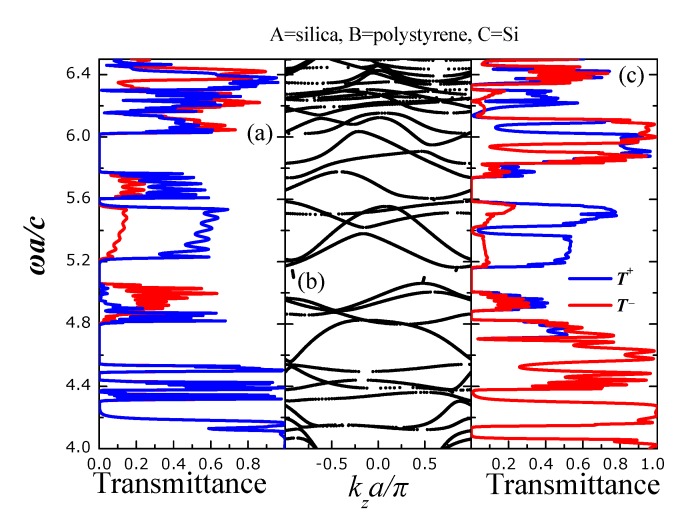
Transmittance for s- (a) and p- (c) polarized light incident with **k**_||_ = (0.25, 0)π/*a* on a finite slab of the PC (see Figure 2) consisting of eight unit layers whereas type-A spheres we have considered silica SiO_2_ (ϵ = 2.1) spheres, as type-B polystyrene (ϵ = 2.6) spheres and as type-C silicon (ϵ = 11.9) (Si) spheres. *T*^+^ (*T*^−^) is the transmittance for light incident from the left (right) (001) faces of the slab. (b) Frequency band structure of the infinitely periodic PC of Figure 1 for **k**_||_= (0.25, 0)*π/a*.

*T*^+^ refers to the transmittance of light incident from the left (001) face of the PC slab, *i.e.*, a wave propagating from left to right whereas *T*^−^ refers to the transmittance of light incident from the right (001) face of the PC slab, *i.e.*, a wave propagating from right to left. Figure 3 shows *T*^+^ and *T*^−^ for light incident off-normally on a finite slab of the Figure 2 with wavevector **k**_||_ = (0.25, 0)π/*a*, for s- (a) and p- (c) polarization. The PC slab consists of eight unit layers where each layer contains four square lattices (planes) arranged as shown in Figure 2. Evidently, there are spectral regions where *T*^+^ and *T*^−^ differ significantly, such as, e.g., from ω*a/c* = 4.8 − 5.0 and from ω*a/c* = 5.2 − 5.8 for *s*-polarized light. For *p*-polarized light, *T*^+^ and *T*^−^ differ substantially from ω*a/c*= 5.9 − 6.1. Despite the important differences in light transmission for left and right incidence a clear case where there is a frequency gap for only one case of incidence direction (left or right) cannot be observed. By inspecting the corresponding frequency band structure (Figure 3b) we see that there exists a single band gap (from ω*a/c* = 5.0 − 5.2) for both polarizations and directions of incidence (left and right) as well as other gaps for only *s*- or *p*-polarized incident light. We also observe a definite asymmetry in the frequency bands relative to the *k_z_* = 0 which stems from the lack of reflection symmetry at this crystal direction.

**Figure 4:**
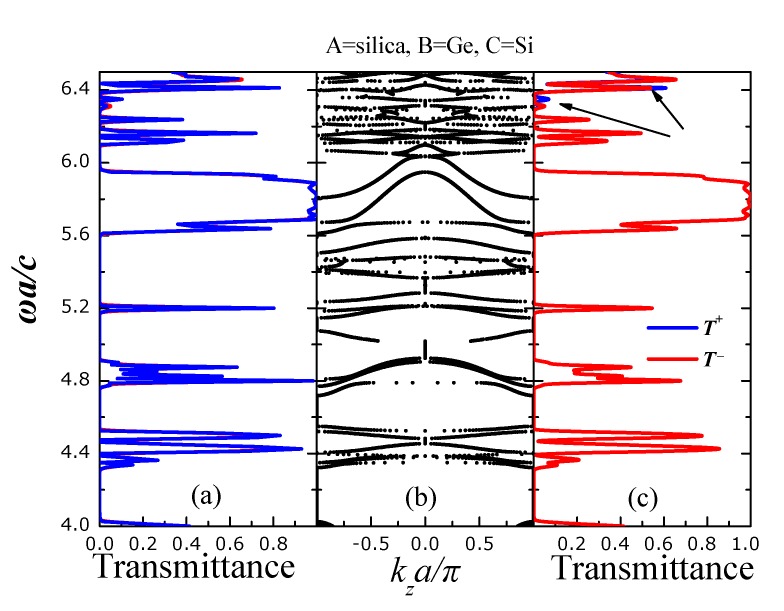
Transmittance for s- (**a**) and p- (**c**) polarized light incident with **k**_||_ = (0.25, 0)π/*a* on a finite slab of the photonic crystal (PC) (see Figure 2) consisting of eight unit layers whereas type-A spheres we have considered silica SiO_2_ (ϵ = 2.1) spheres, as type-B germanium (ϵ = 16.2) spheres and as type-C silicon (ϵ = 11.9) (Si) spheres. *T*^+^ (*T*^−^) is the transmittance for light incident from the left (right) (001) faces of the slab; (**b**) Frequency band structure of the infinitely periodic PC of Figure 1 for **k**_||_ = (0.25, 0)π/*a*.

Next, we consider a PC heterostructure with the (same) unit cell of Figure 1 but with the polystyrene spheres (the B-type spheres in Figure 3) substituted with germanium (*ϵ* = 16.2) ones. Evidently, both spectra of *T*^+^ and *T*^−^ practically coincide apart from two very narrow regions denoted by the arrows in Figure 4c. This is in accordance with the corresponding frequency band structure of Figure 4b where all frequency bands are symmetric with respect to the center *k_z_* = 0. Obviously, the inclusion of materials of high-index contrast in the considered photonic-crystal heterostructures does not favor the emergence of unidirectional propagation.

**Figure 5:**
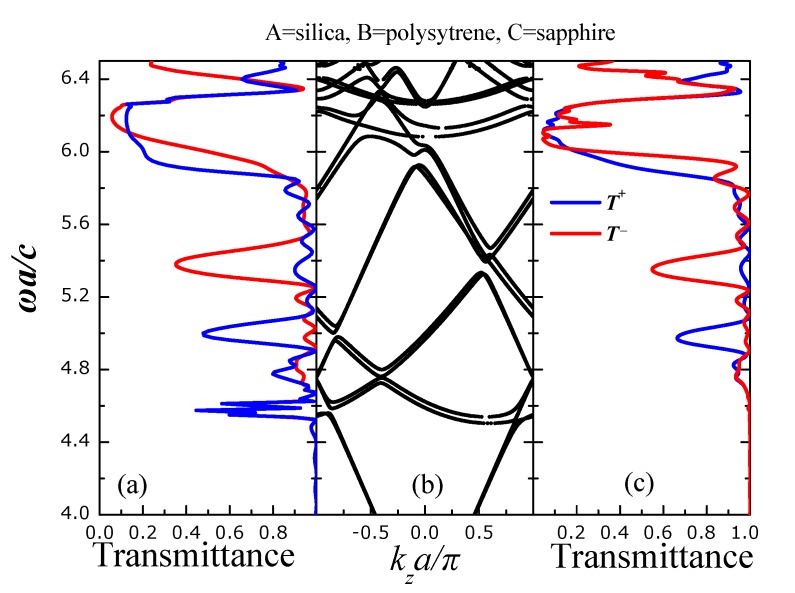
Transmittance for s- (**a**) and p- (**c**) polarized light incident with **k**_||_ = (0.25, 0)π/*a* on a finite slab of the PC of Figure 1 consisting of eight unit layers whereas type-A spheres we have considered silica SiO_2_ (ϵ = 2.1) spheres, as type-B polystyrene (ϵ = 2.6) spheres and as type-C sapphire (ϵ = 3.13) (Si) spheres. *T*^+^ (*T*^−^) is the transmittance for light incident from the left (right) (001) faces of the slab; (**b**) Frequency band structure of the infinitely periodic PC of Figure 1 for **k**_||_ = (0.25, 0)π/*a*.

In an opposite trend (in relation with Figure 4), we substitute the high-index silicon spheres (type-C spheres) of Figure 4 with sapphire (ϵ = 3.13) ones [37]—see Figure 5. We clearly see an entirely different picture from that of Figure 4. The frequency bands show a clear asymmetry with respect to *k_z_* = 0 which is reflected in the corresponding transmission spectra of Figure 5a,c. Namely, we identify two regions of clear unidirectional frequency gaps. Namely, for both *s*- and *p*-polarized light, there exists a *T*^+^-frequency gap around ω*a/c* = 5.0 and a *T*^−^-frequency gap around ω*a/c* = 5.4. Asymmetric transmission (for left- and right- incidence) is also evidenced ω*a/c* = 5.8. We note that the emergence of the unidirectional frequency gaps is not generated by complete absence of frequency bands for one of the *k_z_*-directions (*k_z_* > 0 or *k_z_* < 0). There is a more complex mechanism which explains the emergence of unidirectional frequency gaps in terms of the matching of the group velocities of incident waves with that of the frequency bands (described analytically in [29]).

The  comparison between Figure 4 and Figure 5 leads to the conclusion that the inclusion of high-index spheres does not favor the emergence of unidirectional gaps and propagation. This is a direct consequence of the localized nature of the EM modes of a PC consisting of high-index scatterers. Namely, due to the high refractive index (dielectric constant) of the scatterers the EM field is much localized within a plane of scatterers and wave propagation is achieved by hopping between neighboring planes in a manner resembling the tight-binding picture of electrons in ordinary atomic solids. In accordance with this picture, the lateral (normal to the growth direction of the crystal) displacement of a plane of spheres in an otherwise rectangular arrangement of planes of spheres, see, e.g., the fourth plane of C-type spheres of Figure 1, does not seem to affect seriously the frequency band structure (Figure 4b) and subsequently the transmission spectrum. On the other hand, if the PC comprises of low-index spheres, the photons propagate nearly freely within the PC (a completely opposite picture than the tight-binding mechanism describing wave propagation in a PC of high-index scatterers) except from undergoing diffraction for the *k*-wavevectors implied by the underlying lattice symmetry. Since a monoclinic crystal structure yields an entirely different diffraction pattern than a rectangular one, we expect that the lateral displacement of the fourth plane (of C-type spheres) of Figure 1 to have a large impact on wave propagation of nearly free photons.

## 5. Conclusions

We have shown numerically that it is possible to obtain unidirectional EM wave propagation with plain, linear, dielectric photonic crystals consisting of colloidal micro- or nanospheres without applying external magnetic fields or using nonlinear materials. Prerequisites for observing unidirectional wave propagation are the low symmetry of the photonic crystals and the use of a heterostructure of photonic crystals of different scatterers (being different in material composition and/or size) as the unit cell. We have explored photonic crystals of different material composition wherein we have found that efficient unidirectional propagation in the form of unidirectional frequency gaps/bands emerges when scatterers of low-index contrast are used within the heterostructured unit cell of the crystal. For a practical realization, say, in the optical regime, of a low-symmetric photonic crystal such as those considered here, structures with lattice constant of about 480 nm are needed for a unidirectional band gap around 600 nm, which are usually fabricated via colloidal self-assembly [38,39,40,41].
